# Practical Notes on Diagnosis and Treatment

**Published:** 1906-11-10

**Authors:** 


### Practical Notes on Diagnosis and Treatment.

**Treatment of Infantile Diarrhœa.**

Lieut.-Col. G. H. Younge recommends, after a preliminary dose of castor oil, the use of infusion of quassia as an enema.

**Pepsin.**

The difficulty is to get an active preparation; that known as ingluvin has certainly given me good results in some cases.—*Dr. Robt. Hutchison.*

**Prognosis in Aortic Regurgitation.**

Aortic regurgitation occurring early in life from a rheumatic lesion is, *cæteris paribus*, very much less serious than a similar lesion arising secondary to degeneration of the aorta.—*Sir James Barr.*

**Amyl Nitrite in Migraine.**

As migraine is generally connected with vascular spasm I employed the nitrite of amyl in headache, and found that frequently, though not invariably, it relieved the pain.—*Sir Lauder Brunton.*

**Urinary Calculus and the x-Rays.**

"There are two ways in which the rays may be used: first for screen examination, and second for photographic effect; and beyond question the *former* is the one of service in diagnosing lithiasis." *Mr. Edward W. H. Shenton.*

**Angina Pectoris.**

In most cases, if not in all, the essential factor in an attack of angina pectoris is an increase of peripheral resistance due to exaggerated vaso-constriction. Nitrite of amyl terminates the paroxysm by promoting sudden peripheral vaso-dilation.

**Arterio-Sclerosis.**

In a study of 400 consecutive cases Dr. Brooks found that the coronary arteries are the vessels most affected with arterio-sclerosis; after these come the cerebral vessels, and next those of the kidney. Alcohol seems to be the principal cause of the vascular degeneration.

**The Iodides.**

Sodium iodide is less likely than the potassium salt to interfere with digestion, and is quite as efficacious. For hypodermic administration iodipin is useful, notably in syphilitic disease of the nervous system and in all chronic inflammatory conditions. It is more intensive, and requires less frequent administration.—*Dr. Jas. Burnet.*

**Digitalin in Addison's Disease.**

In Addison's disease the blood pressure may be extremely low. Hence on theoretical grounds digitalin has been employed, and the clinical results show distinct improvement in the symptoms.

**Probilin in Cholelithiasis.**

Probilin is composed of acid sodium oleate, salicylic acid, menthol, and phenolphthalein. Bauermeister reports very favourably of its employment after an experience extending over three years.

**Hand Disinfection.**

To render the hands aseptic Dr. H. J. Boldt recommends that after the nails have been cut short the hands should be scrubbed for five minutes with a good soft soap and in hot water, which should be repeatedly changed. Then for a further period of three to five minutes they should be scrubbed with a 1.5 to 3.0 per cent. solution of veroform.

**Substitutes for Potassium Iodide.**

When with evidences of tertiary syphilis the patient cannot tolerate potassium iodide the position is not an easy one. But it will usually be found in such cases that mercury will act beneficially. One suggestion offered is a dose of one drachm of liquid extract of sarsaparilla thrice daily and an intramuscular injection of mercurial cream, ♏ v once a week. Other authorities give small doses of the iodide with solution of perchloride of mercury. Further alternatives are syrup of hydriodic acid and syrup trifolium, the latter being prepared by Parke, Davis and Co.

**Thyroid Gland in Obesity.**

Mr. W. J. Hoylin finds that thyroid gland has little or no influence on the weight of young persons of either sex. But in females between 25 and 45 years weight rapidly decreases; with males of similar ages the results are uncertain, and only in a proportion is weight permanently reduced. Of 78 females treated, 69 were between 25 and 45 years, their average weekly loss was from 2½ to 4 lbs., and the result was permanent cure. The dose for the first week was 2½ grs. thrice daily; afterwards 5 grains. A "tabloid," after being crushed, was mixed with water or food. Alcohol was forbidden, but no other alteration was made in the diet.

